# Density Functional Theory Study of Methylene Blue Demethylation as a Key Step in Degradation Mediated by Reactive Oxygen Species

**DOI:** 10.3390/ijms26041756

**Published:** 2025-02-19

**Authors:** Silvia González, Ximena Jaramillo-Fierro

**Affiliations:** Departamento de Química, Facultad de Ciencias Exactas y Naturales, Universidad Técnica Particular de Loja, París s/n y Praga, Loja 110107, Ecuador; sgonzalez@utpl.edu.ec

**Keywords:** methylene blue, reactive oxygen species, advanced oxidation processes, photocatalysis, density functional theory, wastewater treatment

## Abstract

Methylene blue (MB), a widely used organic dye, poses significant environmental challenges due to its stability and persistence in aquatic ecosystems. This study employs density functional theory (DFT) to investigate the demethylation mechanisms of MB mediated by reactive oxygen species (ROS), a critical initial step in its photocatalytic degradation. Computational analyses reveal that demethylation is energetically favorable, particularly when mediated by hydroxyl radicals (^•^OH) and hydroxyl ions (OH^−^) with reaction energies of −154 kcal/mol and −214 kcal/mol, respectively. These pathways lead to the formation of key intermediates, such as Azure B, methanol (CH_3_OH), and formaldehyde (CH_2_O), which align with experimentally detected degradation byproducts. The study further demonstrates that the dissociation of hydrogen peroxide species (H_2_O_2_, H_2_O_2_^−^, H_2_O_2_^+^) plays a fundamental role in generating the ROS required for MB degradation. Potential energy surface analyses confirm that these ROS-driven processes are thermodynamically and kinetically viable. The findings provide a theoretical framework that bridges existing knowledge gaps in MB degradation, reinforcing the role of ROS in advanced photocatalytic systems and contributing to the optimization of wastewater treatment strategies. This work underscores the importance of integrating computational and experimental approaches to develop more effective strategies for the remediation of recalcitrant pollutants in wastewater.

## 1. Introduction

**Figure 1:**
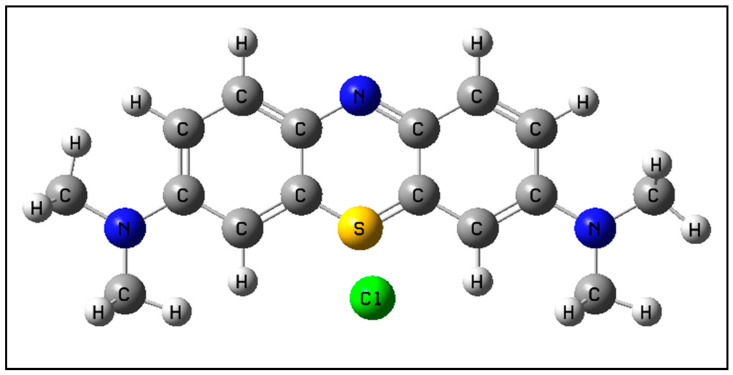
Methylene blue molecular structure.

Methylene blue (MB), a phenothiazine-derived dye, presents a molecular structure characterized by a conjugated aromatic ring system with methyl groups attached to ammonium nitrogen. Figure 1 presents the molecular structure of this species. Its wide use in the textile and chemical industries has generated significant environmental challenges, such as unsightly pollution and eutrophication of water bodies due to effluents containing this dye. MB represents a significant environmental risk, as it contributes to the reduction of photosynthetic activity and biodiversity in aquatic ecosystems [1]. Its conjugated aromatic structure contributes to its high chemical stability and resistance to biodegradation, with its low biological oxygen demand/chemical oxygen demand (BOD/COD) ratio complicating treatment efforts [2]. The persistence of MB in aquatic environments underlines the classification of this dye as a priority pollutant [3,4], thus requiring effective degradation methods to mitigate its ecological impact.

Advanced oxidation processes (AOPs), in particular heterogeneous photocatalysis, have been shown to be highly effective in the degradation of recalcitrant pollutants such as MB [5,6,7]. MB photodegradation relies on reactive oxygen species (ROS), such as hydroxyl radicals (^•^OH), superoxide ions (O_2_^•−^), hydrogen peroxide (H_2_O_2_), and singlet oxygen (^1^O_2_), which are generated when photons are absorbed by photocatalytic materials [8]. This process excites electrons from the valence band (VB) to the conduction band (CB), forming electron–hole pairs (e^−^/h^+^) that interact with water or oxygen to produce ROS [9]. These species initiate oxidative reactions, leading to the rapid decomposition and complete mineralization of organic compounds [10]. The dynamics of these species, especially on the surface of photocatalysts such as TiO_2_, play a fundamental role in the initiation of oxidative degradation processes. Although ROS can be generated directly by photocatalysts, numerous studies have demonstrated that H_2_O_2_ plays a critical role in enhancing oxidation processes by acting as a precursor for hydroxyl radicals (^•^OH). In many advanced oxidation processes (AOPs), the decomposition of H_2_O_2_ into ROS accelerates MB degradation [1,11]. The present study focuses on H_2_O_2_-mediated ROS formation as a representative mechanism for MB oxidation, providing a thermodynamically validated stepwise degradation model that complements experimental observations of degradation intermediates.

Recent advances in photocatalysis have focused on the development of visible and UV-active catalysts, such as those based on TiO_2_ [12,13], ZnO [14,15], TiO_2_/ZnO [16,17], ZnTiO_3_/TiO_2_ [18,19], nano-catalysts stabilized with natural surfactants [20], and other nanoparticle-based photocatalysts [21], which have demonstrated remarkable potential in the degradation of recalcitrant organic pollutants, such as methylene blue (MB). Similarly, nanocomposites such as h-MoO_3_, P2ABSATiO_2_, and others have demonstrated efficacy in producing ROS when exposed to visible light, highlighting the importance of proper material design [22,23,24]. On the other hand, catalysts such as Nb_2_O_5_ and ZnO, known for their ability to generate highly oxidative peroxo species in the presence of H_2_O_2_, have also shown high efficiency in MB degradation [25,26]. The properties of these photocatalysts, such as their surface area and electronic structure, play a fundamental role in ROS production, underlining the urgent need to develop new materials that further optimize ROS generation and improve the overall performance of photocatalytic systems.

Reactive oxygen species (ROS), such as hydroxyl radicals (^•^OH) and superoxo species (O_2_^•−^), play a critical role in MB degradation by initiating bond-breaking reactions [27]. Hydrogen peroxide, although less directly involved, acts as a critical precursor for ^•^OH radicals under appropriate catalytic conditions, highlighting the interaction between several ROS in the degradation process [3,25,28]. The interaction between ROS and MB drives a series of degradation pathways, in particular demethylation and central ring opening, which fundamentally alter the molecular constitution of the dye and lead to its decomposition into simpler inorganic compounds.

Among the different ROS involved in MB degradation, hydroxyl radicals (^•^OH), protons (H^+^), and hydroxyl ions (OH^−^) play a pivotal role in the oxidative cleavage of the methyl (–CH_3_) groups attached to the nitrogen atoms in MB. Hydroxyl radicals, possessing a high oxidation potential (~2.8 V vs. SHE), initiate the degradation process by abstracting hydrogen atoms from the methyl groups, leading to stepwise demethylation and the formation of intermediates such as Azure B, Azure A, and Azure C [29]. The equilibrium between OH^−^ and ^•^OH in alkaline conditions enhances radical formation, facilitating efficient oxidation, while in acidic environments, H^+^ ions contribute to the generation of hydrogen peroxide (H_2_O_2_), a precursor of ^•^OH radicals [10]. Experimental studies suggest the presence of key degradation byproducts such as methanol (CH_3_OH) and formaldehyde (CH_2_O), supporting the hypothesis that demethylation occurs prior to the cleavage of the aromatic rings, a process influenced by bond energy considerations [2,30]. Although hydroxyl radicals are essential in demethylation, their main involvement occurs in advanced stages of degradation, such as aromatic ring cleavage and hydroxylation [11,31]. Demethylation results in the formation of other key intermediates besides azures, including thionine and a variety of organic acids and alcohols, which are subsequently subjected to hydroxylation. This process facilitates the cleavage of aromatic rings, culminating in the complete mineralization of MB into inorganic products such as CO_2_, H_2_O, NH_4_^+^, and SO_4_^2−^ [27,31,32].

Although several catalysts have shown considerable potential for environmental remediation by breaking down MB, the underlying mechanisms of molecular degradation of this dye remain a matter of debate. A central aspect of this discussion is the ambiguity regarding the initial step in the degradation pathway: some studies suggest cleavage of the central aromatic ring, while others propose that degradation begins with methyl substituents on the benzene ring [11,25,32]. This discrepancy points to a significant gap in the experimental validation of the initial degradation mechanisms of MB, particularly the role of demethylation. Hydroxyl radicals are known to hydroxylate aromatic structures, leading to the opening of the central aromatic heterocycle and the subsequent formation of smaller organic fragments [31]. However, the involvement of methyl substituents on the benzene ring is unclear. Demethylation as an initial step in the degradation pathway remains unexplored. While theoretical studies have proposed that progressive oxidation of dimethyl-phenyl-amino groups may involve demethylation [3,27], direct experimental evidence for this process is lacking.

Advanced analytical techniques, such as LC-MS, FTIR, and MALDI-TOF, have revealed the complexity of methylene blue (MB) degradation, which involves sequential oxidative transformations [10,30,33]. These methods enable real-time monitoring of transformations, providing critical insights into the degradation pathways [11,30]. Theoretical models support the role of bond dissociation energies in guiding these reactions, while the analytical techniques confirm the formation of intermediates that showcase the interactions between ROS and MB molecules [2,31,32,33]. Despite these advancements, explicit experimental validation of demethylation as the definitive starting point remains limited, leaving a critical gap in understanding the initial stages of MB degradation and highlighting the need for further studies to refine theoretical models and confirm these pathways [27,32]. Addressing the knowledge gap in methylene blue (MB) degradation mechanisms is critical for advancing our understanding and optimizing photocatalytic systems. If demethylation is indeed the initial step in MB degradation, it represents a pivotal target for the design of more efficient catalysts.

Computational methods, particularly density functional theory (DFT), provide a powerful tool for exploring these mechanisms at the molecular level [34,35]. DFT facilitates the analysis of reaction energetics, transition states, and potential intermediates, offering a robust theoretical framework to complement and enhance experimental findings [36,37]. This study focuses on investigating the demethylation of MB mediated by reactive oxygen species (ROS) using computational DFT methods to elucidate the degradation pathway. By examining reaction energetics and identifying key intermediates, the research aims to assess the feasibility of demethylation as the initiating step in MB degradation. Through theoretical modeling, this work seeks to clarify the role of demethylation, address existing knowledge gaps, and contribute to the development of more efficient photocatalytic strategies for environmental remediation. Although experimental studies have identified MB degradation intermediates using analytical techniques such as mass spectrometry, FTIR, and UV–vis, no prior research has explicitly elucidated the molecular steps leading to their formation. The absence of a thermodynamically supported, stepwise degradation mechanism motivated this study, which provides a first-principles computational framework to clarify MB demethylation. By integrating theoretical insights with experimentally detected intermediates, this work establishes a mechanistic foundation for ROS-mediated MB degradation.

## 2. Results

**Figure 2:**
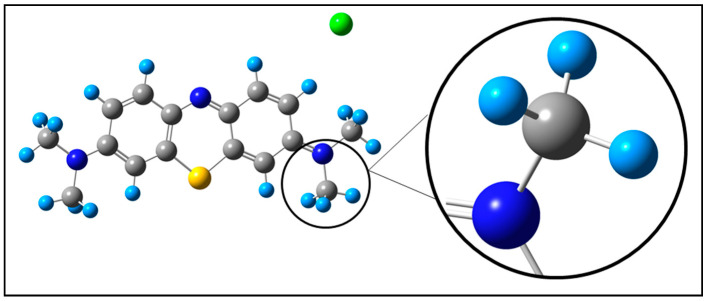
The methylene blue model used in this study, with an enlarged detail of the terminal methyl group. The gray balls are C atoms, the small light blue balls are H atoms, the green ball is a chlorine ion, the dark blue balls are N atoms, and the yellow ball is an S atom.

MB has a stable, heterocyclic, highly symmetric molecular structure, as shown in Figure 2. The decomposition of this molecule requires complex reactions, with the formation and breaking of several bonds, with parallel and consecutive reactions and with the presence of various radicals, ions, and species in a solution. Demethylation, as the first step in the decomposition of MB, causes the loss of symmetry in the molecule, leaving it more exposed to reactions.

### 2.1. ROS Formation from H_2_O_2_ Species Dissociation

**Figure 3:**
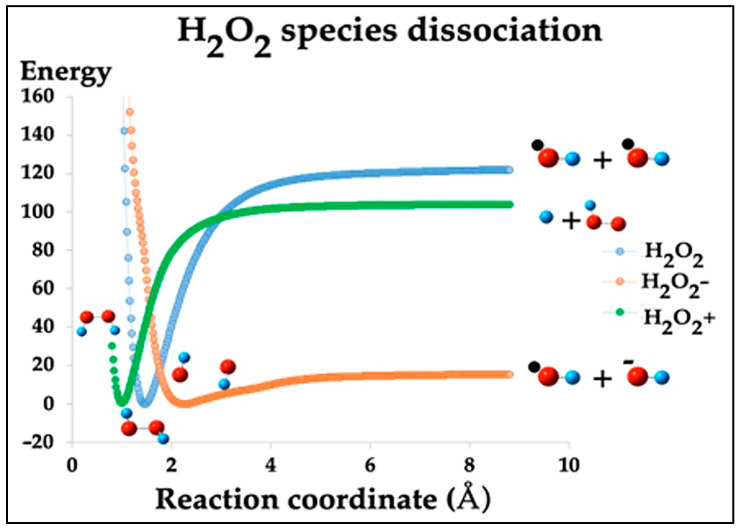
Potential energy surface of the dissociation of the three species of H_2_O_2_ and their respective products. The units of energy are kcal/mol. In 0 kcal/mol, each molecule has an equilibrium molecular distance.

Hydrogen peroxide (H_2_O_2_) is a mild oxidizing agent capable of oxidizing a wide range of organic and inorganic compounds. It is notable for being the only stable reactive oxygen species (ROS), allowing its detection after the decomposition of other reactive species in various photochemical and catalytic processes [38,39]. Under specific energy conditions, H_2_O_2_ can undergo ionization, generating its charged forms: the H_2_O_2_^+^ ion, produced by the removal of an electron, giving rise to a positively charged species, and the H_2_O_2_^−^ ion, formed when H_2_O_2_ acquires an additional electron, giving rise to a negatively charged species. These transformations occur in high-energy environments, such as in photoelectron spectroscopy experiments or chemical reactions involving highly oxidizing reactive species [40,41]. In this study, since specific reactive oxygen species (ROS) are required to attack the methyl group of the MB molecule, particularly ^•^OH, H^+^, and OH^−^, these are initially generated from the dissociation of H_2_O_2_, H_2_O_2_^+^, and H_2_O_2_^−^. Dissociation of H_2_O_2_ produces hydroxyl radicals (^•^OH), dissociation of H_2_O_2_^−^ generates hydroxyl ions (OH^−^), and dissociation of H_2_O_2_^+^ produces protons (H^+^). Table 1 summarizes the results of these three dissociation reactions, while Figure 3 illustrates the potential energy surfaces associated with these reactions. For reference, dissociation of H_2_ is also included.

### 2.2. Demethylation of Methylene Blue

This study reports ROS-mediated MB demethylation, exploring possible reaction pathways. The selection of the demethylation pathways investigated in this study was based on experimental and theoretical considerations. Previous studies on MB photodegradation have identified intermediates such as Azure A, Azure B, Azure C, and thionin (CH_2_O) using mass spectrometry and spectroscopic techniques [31], but the stepwise molecular transformations leading to these products remain unclear. To address this gap, the present study systematically examines three plausible ROS-mediated demethylation pathways involving hydroxyl radicals (OH), hydroxyl ions (OH^−^), and protons (H^+^). These species were chosen due to their known reactivity in advanced oxidation processes (AOPs) and their critical role in oxidative degradation mechanisms. The goal is to evaluate whether these pathways are thermodynamically viable and whether they provide a molecular-level explanation for the formation of key MB degradation intermediates. Finally, the reaction pathways were inferred from free energy differences between reactants and products, providing a thermodynamic perspective on ROS-mediated demethylation.

#### 2.2.1. Pathway 1: Demethylation of MB with H^+^

The source of the hydrogen cation (H^+^) is unlikely to be molecular hydrogen (H_2_) due to several considerations. First, in a photocatalytically active environment, H_2_ has not been identified as a significant contributor. Second, the simultaneous presence of H^+^ cations and hydroxyl radicals (^•^OH) or hydroxyl ions (OH^−^) would readily form water (H_2_O), reducing the availability of H^+^ for reaction. A more plausible source of H^+^ in a photoactive environment with ROS is H_2_O_2_^+^, as its dissociation generates both the H^+^ cation and the ^•^OOH radical, which have been experimentally detected in MB degradation processes.

Furthermore, the dissociation of H_2_O_2_^+^ requires slightly less energy (104 kcal/mol) compared to the dissociation of H_2_ (110 kcal/mol), reinforcing the likelihood of H_2_O_2_^+^ as the H^+^ source. The ^•^OOH radical, a byproduct of H_2_O_2_^+^ dissociation, is commonly associated with MB degradation, providing additional evidence for this mechanism.

**Figure 4:**
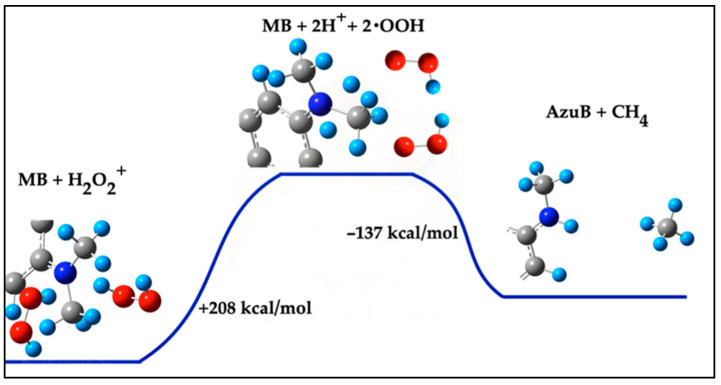
Demethylation of MB with two H^+^ cations from the dissociation of two H_2_O_2_^+^ molecules, and the formation of Azure B and CH_4_.

The products of this demethylation reaction are Azure B (MB with one CH_3_ group replaced by a hydrogen atom) and methane (CH_4_). Obtaining one H atom from H_2_O_2_^+^ requires 104 kcal/mol, and since two H atoms are necessary for this reaction, the total energy required is 208 kcal/mol. As shown in Figure 4, two H^+^ cations interact with the MB molecule, breaking the N–CH_3_ bond. One H^+^ cation binds to the MB molecule, forming Azure B, while the other H^+^ reacts with the detached CH_3_ group to produce CH_4_. The overall reaction energy from MB + 2 H_2_O_2_^+^ to Azure B + CH_4_ is calculated to be +71 kcal/mol.

#### 2.2.2. Pathway 2: Demethylation of MB with OH^−^ Forming CH_3_OH

**Figure 5:**
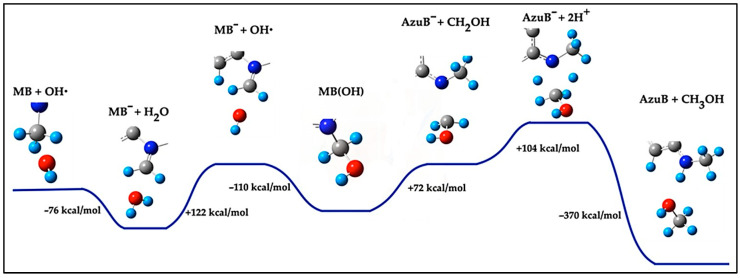
Pathway reaction to demethylation of MB with two ^•^OH radicals to form Azure B and CH_3_OH.

A plausible pathway for the demethylation of methylene blue (MB) involves hydroxyl species, specifically hydroxyl radicals (^•^OH) or hydroxyl ions (OH^−^). In the first step, one of these species attacks an external methyl group (CH_3_^−^) of the MB molecule, abstracting a hydrogen atom (H) to form a water molecule (H_2_O). This reaction is highly exothermic, releasing −76 kcal/mol, as shown in Figure 5. This value is consistent with reported hydrogen abstraction energies for organic and inorganic systems [42,43,44]. The high exothermicity observed in this reaction underscores the strong thermodynamic driving force for MB degradation, supporting the effectiveness of hydroxyl radicals in photocatalytic and environmental remediation applications. It is important to note that whether the reactive species is ^•^OH or OH^−^, the reaction generates the same intermediate structure (MB^−^), where the methyl group becomes deprotonated and more susceptible to further oxidation:

H_2_O_2_ → 2 ^•^OH; E = 122 kcal/mol (1)

MB + ^•^OH → MB^−^ + H_2_O; E = −76 kcal/mol (2)

This intermediate (MB^−^) subsequently reacts with another ^•^OH radical, derived from H_2_O_2_ dissociation, which involves an energy cost of 122 kcal/mol. The ^•^OH radical adds to the desaturated carbon of the methyl group, converting it into a –CH_2_–OH group (MB(OH)) with an energy release of −110 kcal/mol:

MB^−^ + ^•^OH → MB(OH); E = −110 kcal/mol (3)

The final step in the demethylation process involves breaking the N–C bond linking the –CH_2_–OH group to the MB molecule. This step requires 72 kcal/mol and results in the formation of Azure B (AzuB^−^) and a –CH_2_OH fragment:

MB(OH) → AzuB^−^ + CH_2_OH; E = 72 kcal/mol (4)

The –CH_2_OH fragment reacts further to form methanol (CH_3_OH), while the nitrogen terminal in AzuB^−^ is activated to accept a cation (H^+^), forming Azure B. These two cations (H^+^) are sourced from the dissociation of two H_2_O_2_^+^ molecules, each requiring 104 kcal/mol:

2H_2_O_2_^+^ → 2 H^+^ + 2 ^•^OOH; E = 208 kcal/mol (5)

The final reaction for the formation of Azure B and methanol is highly exothermic, releasing −370 kcal/mol:

AzuB^−^ + CH_2_OH + 2 H^+^ → AzuB + CH_3_OH; E = −370 kcal/mol (6)

Applying Hess’s Law, the total energy of this reaction is the sum of all steps, resulting in an overall exothermic reaction:

ΔEtotal = +122 kcal/mol − 76 kcal/mol − 110 kcal/mol + 72 kcal/mol + 208 kcal/mol − 370 kcal/mol = −154 kcal/mol(7)

This indicates that the reaction is energetically favorable, despite requiring energy input for key steps, such as the dissociation of H_2_O_2_ to produce ^•^OH radicals, the cleavage of the N–C bond, and the dissociation of H_2_O_2_^+^ molecules.

Although the total energy calculation confirms the exothermic nature of the reaction, the mechanism involves energy-intensive steps. These steps are plausible in a photoactive medium where a photocatalyst facilitates energy absorption and enhances ROS generation. The identification of methanol (CH_3_OH) as a byproduct in experimental studies further supports the validity of this pathway, demonstrating its role in MB degradation under photocatalytic conditions. Optimizing photocatalysts for higher efficiency in ROS production could make this pathway even more effective, providing a practical solution for environmental remediation.

#### 2.2.3. Pathway 3: Demethylation of MB with OH^−^ Forming CHOH

To obtain formaldehyde (CHOH) through the demethylation of methylene blue (MB), two possible mechanisms are proposed. Both mechanisms share the initial steps with Pathway 2 up to the point where the fragments AzuB^−^ and –CH_2_OH are formed after breaking the N–C bond. Pathways 3 involves H_2_O_2_ and/or its ionic species. Hydrogen peroxide (H_2_O_2_) is a moderate oxidizing agent and the only stable reactive oxygen species (ROS) detectable after the decay of other ROS. Numerous studies have demonstrated that H_2_O_2_ plays a critical role in enhancing oxidation processes by acting as a precursor for hydroxyl radicals (^•^OH) [38,39]. The ionic forms of H_2_O_2_, H_2_O_2_^+^, and H_2_O_2_^−^ are generated through the loss or gain of an electron and can be formed in reactions involving reactive species [40,41]. Although these species have been relatively poorly studied, they may play an important role in photocatalytic processes. In photocatalytic settings, H_2_O_2_^+^ dissociation serves as a potential source of protons (H^+^), contributing to oxidative degradation pathways. However, it is not the only source of H^+^, as protons are naturally abundant in aqueous environments due to water autoionization and external pH conditions [45]. The role of H_2_O_2_^+^ in this context is to act as an additional contributor to proton availability, particularly in oxidative systems where peroxo species participate in the redox cycle. Therefore, the contribution of H_2_O_2_^+^ to H^+^ availability should be considered as one of several possible mechanisms rather than the exclusive pathway.

##### Pathway 3a: Reaction with H_2_O_2_

In this mechanism, the AzuB^−^ and –CH_2_OH fragments react with a molecule of H_2_O_2_. The N terminal of AzuB^−^ is activated and interacts with H_2_O_2_, resulting in the formation of Azure B and formaldehyde (CHOH). This process involves an energy of +8 kcal/mol for the steps leading to AzuB^−^ and –CH_2_OH formation, followed by the reaction of these fragments with H_2_O_2_, which releases −160 kcal/mol. Hence, the total energy for this pathway is calculated as −152 kcal/mol:

AzuB^−^ + CH_2_OH + H_2_O_2_ → AzuB + CHOH; E = −160 kcal/mol (8)

**Figure 6:**
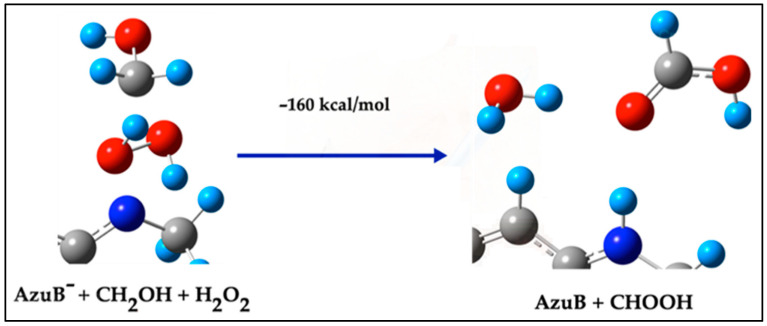
Step reaction to form directly Azure B and CHOH from AzuB^−^ + –CH_2_OH.

This reaction is shown in Figure 6.

The self-consistent field (SCF) optimization confirms that the interaction of the activated N terminal and –CH_2_OH with H_2_O_2_ forms Azure B and formaldehyde. Formaldehyde has been experimentally identified in MB degradation, supporting the plausibility of this reaction pathway [3,32].

##### Pathway 3b: Reaction Involving H_2_O_2_^+^, H_2_O_2_^−^, and H_2_O_2_

This alternative mechanism begins with the interaction of AzuB^−^ and –CH_2_OH with H_2_O_2_^+^. The dissociation of H_2_O_2_^+^ generates an ^•^OOH radical and an H^+^ cation. The ^•^OOH radical attacks the –CH_2_OH fragment, forming an intermediate species (CH_2_(OH)_2_) and an ^•^O radical, while the H^+^ activates the N terminal to form Azure B:

AzuB^−^ + CH_2_OH + H_2_O_2_^+^ → AzuB + ^•^O + CH_2_(OH)_2_; E= −260 kcal/mol (9)

**Figure 7:**
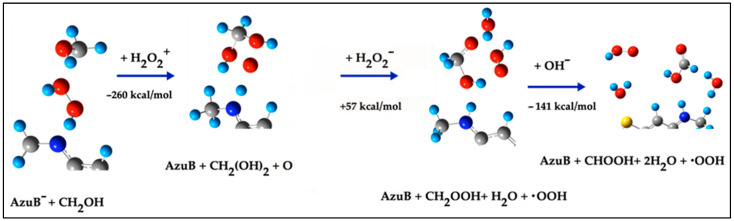
Step reaction to form Azure B and CHOH from AzuB^−^ + −CH_2_OH, involving H_2_O_2_^+^, H_2_O_2_^−^ and H_2_O_2_.

This mechanism is shown in Figure 7.

Subsequently, the interaction of Azure B, ^•^O, and CH_2_(OH)_2_ with H_2_O_2_^−^ generates CH_2_OOH, Azure B, and an H_2_O molecule:

AzuB + ^•^O + CH_2_(OH)_2_ + H_2_O_2_^−^ → AzuB + CH_2_OOH + H_2_O; E = 57 kcal/mol (10)

Finally, CH_2_OOH reacts with an ^•^OH radical (from H_2_O_2_ dissociation) to form formaldehyde and two H_2_O molecules:

H_2_O_2_ → 2 ^•^OH; E = 122 kcal/mol (11)

AzuB + CH_2_OOH + H_2_O + ^•^OH → AzuB + CHOH + 2H_2_O; E = −141 kcal/mol (12)

The energy for these combined steps totals −214 kcal/mol, making this pathway more energetically favorable than Pathway 3a.

**Figure 8:**
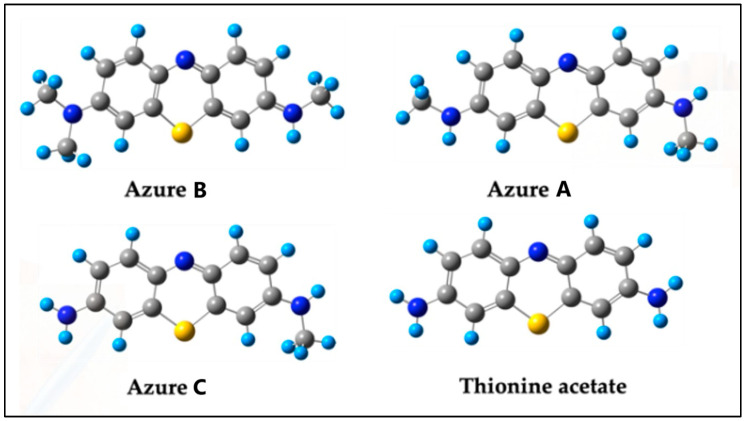
Transformation of the MB for demethylation to thionine acetate.

Finally, considering the substitution of the two methyl fragments to obtain Azure A, and that the final species will be thionine acetate, the total necessary energy for the full demethylation could be 4 × −214 kcal/mol = −856 kcal/mol, as is shown in Figure 8.

The total demethylation energy was estimated by multiplying the single-step energy by four, assuming that all CH_3_ groups exhibit similar reactivity. While successive demethylation steps may exhibit slight variations due to electronic stabilization effects, this approach provides a first-order approximation of the overall energy requirement.

## 3. Discussion

In this study, Pathway 1 involves the interaction of H^+^ cations, derived from H_2_O_2_^+^ dissociation, with the methyl group of methylene blue (MB). Although this mechanism is theoretically feasible, it requires significant energy input, with a total reaction energy of +71 kcal/mol. The formation of Azure B and methane (CH_4_) suggests a straightforward demethylation process. However, the high energy cost, combined with the simultaneous presence of ^•^OH and H^+^ potentially forming water (H_2_O), makes this pathway less likely under typical photocatalytic conditions [31]. Its reliance on H_2_O_2_^+^ as a cation source further limits its efficiency in diverse environments. On the other hand, Pathway 2 represents a more energetically favorable mechanism than Pathway 1, with a total energy of −154 kcal/mol. It begins with the attack of an OH^−^ ion or ^•^OH radical on the methyl group of MB, forming methanol (CH_3_OH) and Azure B. The initial steps, including H abstraction and N–C bond cleavage, are highly exothermic, releasing −76 kcal/mol and −110 kcal/mol, respectively. Experimental observations of methanol as a byproduct support the feasibility of this pathway [2,30]. This mechanism’s efficiency underscores the critical role of hydroxyl species in driving MB degradation.

Regarding Pathway 3, Pathway 3a involves the reaction of AzuB^−^ and –CH_2_OH fragments with an H_2_O_2_ molecule, resulting in the formation of Azure B and formaldehyde (CHOH). The total energy of this pathway is −152 kcal/mol, which is energetically comparable to Pathway 2. The reaction proceeds via activation of the N terminal in AzuB^−^ and oxidation of the –CH_2_OH fragment. While the reliance on H_2_O_2_ as the ROS source is effective, it contrasts with the more efficient use of OH^−^ in Pathway 2. On the other hand, Pathway 3b is energetically more favorable than 3a, with a total reaction energy of −214 kcal/mol. It begins with the dissociation of H_2_O_2_^+^ to produce ^•^OOH radicals and H^+^ cations, facilitating the formation of an intermediate (CH_2_(OH)_2_). The interaction of these intermediates with H_2_O_2_^−^ generates CH_2_OOH, which subsequently reacts with ^•^OH to form formaldehyde (CHOH). This multi-step process highlights the significant contribution of various ROS in driving oxidative transformations [10,46].

Among the four pathways, Pathway 3b emerges as the most probable mechanism due to its lower energy requirement (−214 kcal/mol) and alignment with formaldehyde production proposed by several experimental studies [30,31,32]. The interaction of H_2_O_2_^+^ with the methyl group facilitates rapid and efficient demethylation, making it a dominant pathway under typical photocatalytic conditions. Pathway 2, with a total energy of −214 kcal/mol, is also plausible, especially in environments rich in OH^−^. Its multi-step nature involving the formation of CH_3_OH provides an alternative route for MB degradation. However, its higher energy demand and reliance on multiple ROS species make it less favorable compared to Pathway 3b. Pathway 3a, while simpler, has a similar energy cost (−152 kcal/mol) and depends on the availability of H_2_O_2_. Pathway 1, requiring a total energy of +71 kcal/mol, is the least probable mechanism due to its inefficiency and the high likelihood of water formation when H^+^ cations coexist with hydroxyl species. The complete demethylation of MB to form Azure A and thionine acetate involves the sequential substitution of all methyl groups, with a total energy requirement of approximately −856 kcal/mol. This highlights the cumulative energy savings achieved in photocatalytic environments, where energy input is continuously replenished through photon absorption, enabling such transformations [13,25].

The computational results presented here align well with experimental findings on MB photodegradation. Literature studies have detected intermediates such as Azure B and methanol as dominant degradation products, consistent with the proposed demethylation mechanisms. Additionally, previous computational studies have suggested hydroxyl radical (^•^OH) attack as a primary step in MB degradation, supporting the mechanistic pathways explored in this work. Table 2 summarizes the predicted intermediates from this study alongside experimental data, demonstrating strong agreement between theoretical and observed degradation pathways. While this study does not explicitly model catalyst–ROS interactions, the proposed mechanisms provide a thermodynamic basis for understanding how ROS contribute to MB degradation, complementing existing experimental research.

The results of this study emphasize the critical role of photocatalysts in optimizing ROS production and facilitating demethylation pathways. Catalysts such as sulfur-doped TiO_2_ and h-MoO_3_, which enhance OH^−^ and ^•^OH generation under visible light, could further improve the efficiency of Pathway 3b [12,13]. Future research should focus on validating these mechanisms experimentally using advanced techniques like LC-MS and FTIR to detect intermediates such as methanol and formaldehyde in real time. On the other hand, the environmental factors, including pH, temperature, and ROS concentration, must also be considered to determine the predominance of each pathway. Hybrid photocatalytic systems integrating materials capable of generating multiple ROS species could address the limitations of individual pathways, offering more robust solutions for wastewater remediation. Overall, this study provides a comprehensive understanding of MB degradation mechanisms, highlighting the interplay between ROS and reaction energetics, and lays the groundwork for the development of more efficient and sustainable photocatalytic strategies.

## 4. Materials and Methods

The study of MB demethylation as a key step in degradation mediated by ROS was conducted using density functional theory (DFT) [47]. The Gaussian [48] version 16 software package (Gaussian, Inc., Wallingford, CT, USA) was used to perform calculations using the B3LYP hybrid exchange-correlation functional. The B3LYP exchange-correlation functional was selected for its well-documented capability to balance computational cost with accuracy in predicting molecular properties of ROS species. This hybrid functional incorporates a mix of Hartree–Fock (HF) and density functional theory (DFT) gradients, significantly improving the description of electronic interactions, particularly important in the chemistry of reactive species like ROS. The effectiveness of B3LYP in predicting electronic, geometric, and energetic properties has been validated extensively in the literature, supporting its applicability to the systems studied in our research [49,50,51].

The molecular properties of the species under study were computed using the Ahlrichs et al. basis set, specifically the triple-zeta valence polarization (TZVP) basis set. This basis set is grounded in its proven ability to provide a detailed and accurate electronic structure representation of molecules, a critical aspect when studying ROS chemistry [52,53]. This basis set, optimized to balance the precision of valence orbital description with computational demand, ensures a reliable depiction of critical elements, allowing for accurate predictions of the stability and reactivity of the species studied. The choice of TZVP underpins the reliability of this study, enabling precise insights into the molecular properties of ROS.

In this study, the root mean square convergence criterion for the density matrix in the self-consistent field (SCF) iteration was set to 10^−14^ a.u., aiming for an energy convergence threshold of at least 10–15 a.u. (GAUSSIAN keyword: SCF = tight). This default convergence criteria recommended by Gaussian 16 was applied to ensure the reliability and precision of the calculations. These criteria ensure that the resulting structures represent appropriate energy minima, which is an important aspect for studies aiming to understand the stability and reaction pathways of ROS. The role of ROS was analyzed by modeling their direct interactions with MB and evaluating whether these reactions are energetically favorable. Hydroxyl radicals (^•^OH), hydroxyl ions (OH^−^), and protons (H^+^) were selected as key oxidizing agents based on their established reactivity in oxidation reactions. The energy diagrams in this study were constructed by performing separate calculations for each species involved in MB demethylation. The reaction energies were estimated based on free energy differences between reactants and products. This approach provides a thermodynamic perspective on MB degradation. The total energy, defined here as the sum of all electronic contributions plus zero-point energy corrections, is an important metric to evaluate the thermodynamic stability and chemical reactivity of ROS. The precision in the determination of the total energy allows direct comparisons between different chemical species and the evaluation of possible reaction pathways and mechanisms.

After the geometric optimization of the molecules, which aims to locate the structures at their most stable energy minimum, we proceeded with the calculation of the vibrational frequencies. Vibrational frequency calculations are fundamental for confirming that optimized geometries represent true energy minima, as indicated by the absence of imaginary frequencies. These calculations provide insights into molecular stability and potential reaction mechanisms, enhancing our understanding of ROS dynamics. Secondly, they provide valuable insights into molecular dynamics, allowing for a better understanding of how ROS interact and react in various contexts. The vibrational frequencies were calculated by applying the principle of harmonic oscillation, which assumes that molecular vibrations near equilibrium can be modelled as harmonic oscillators. Gaussian 16 uses advanced algorithms to determine force constants from which frequencies are calculated.

The analysis of vibrational frequencies offers detailed information on the rigidity of molecular bonds and the stability of structures. Higher frequencies indicate stronger bonds and more rigid structures, while lower frequencies may indicate weaker bonds or more flexible molecular groups. This analysis is complemented by the calculation of the zero-point energy (ZPE) and thermal corrections to thermodynamic properties such as enthalpy and Gibbs free energy, which are critical to understanding the thermodynamics of the reactions in which ROS participate. By integrating the Polarizable Continuum Model (PCM), it was possible assess the impact of the solvent environment on vibrational frequencies, which is fundamental for accurate simulations of molecular dynamics in aqueous solutions.

The PCM simulates the solvent as a continuous polarizable medium surrounding the solute, automatically generating a virtual cavity based on the molecular geometry of the study molecule, ensuring an accurate representation of the polarizing effect of the solvent [54]. The selection of water as a solvent in all simulations was based on its relevance in biological and photocatalytic processes in which ROS play an important role. Gaussian 16 allows water to be specified as a solvent by adjusting its dielectric constant (ε = 78.4), together with the surface tension and other relevant solvent parameters, to values characteristic of water at room temperature. These adjustments are essential to align the simulations with real experimental conditions, ensuring that the computational findings are applicable and relevant to the analysis of ROS interactions and reactions under typical experimental conditions.

Finally, visualization of all molecular structures and properties was enabled by the GaussView version 6 software package (Semichem Inc., Shawnee Mission, KS, USA).

## 5. Conclusions

This study provides a comprehensive computational investigation into the demethylation of methylene blue (MB) as a critical initial step in its degradation mediated by reactive oxygen species (ROS). Employing density functional theory (DFT), the energetics, intermediates, and potential pathways were explored to elucidate the molecular mechanisms underlying MB degradation. The findings confirm that the dissociation of hydrogen peroxide species (H_2_O_2_, H_2_O_2_^−^, and H_2_O_2_^+^) plays a fundamental role in generating ROS, such as hydroxyl radicals (^•^OH), which are crucial for initiating the demethylation process. Among the pathways examined, demethylation mediated by hydroxyl radicals and hydroxyl ions was identified as a key mechanism, leading to the formation of intermediates like Azure B, methanol, and formaldehyde. The energetics of these processes highlight the feasibility of demethylation as the initial degradation step, supported by the favorable bond dissociation energies and the experimental identification of key byproducts.

This study also demonstrates the critical role of advanced photocatalytic systems in facilitating ROS production and enhancing degradation efficiency. Insights into the interaction between MB and ROS, including the cleavage of methyl groups and subsequent ring-opening reactions, provide a deeper understanding of the sequential transformations leading to the mineralization of MB into CO_2_, H_2_O, NH_4_^+^, and SO_4_^2^^−^. These results bridge important knowledge gaps by validating demethylation as a plausible initial step in MB degradation, offering a theoretical foundation that complements existing experimental data. The findings underscore the importance of optimizing photocatalytic systems and ROS generation to develop more efficient strategies for environmental remediation.

Future research should focus on experimental validation of these pathways, particularly under various catalytic conditions, and the design of advanced materials to enhance ROS production, paving the way for practical applications in wastewater treatment. This integrated approach combining computational and experimental methodologies holds great potential for advancing the field of photocatalytic degradation of recalcitrant pollutants.

## Figures and Tables

**Table 1:** Dissociation energy of three species of H_2_O_2_ and their respective products.

Origen Species	Products	Dissociation Energy (kcal/mol)
H_2_O_2_	^•^OH + ^•^OH	122
H_2_O_2_^−^	^•^OH + OH^−^	15
H_2_O_2_^+^	H^+^ + ^•^OOH	104
H_2_	H^+^ + H^+^	110

**Table 2:** Comparison of computationally predicted and experimentally observed MB degradation intermediates.

Intermediate	Experimental Studies	Predicted in This Study
Azure A, Azure B, Azure C, Thionin	Detected by LC-MS and LC-UV/Vis	Yes
Methanol (CH_3_OH)	Detected by GC-MS	Yes
Formaldehyde (CH_2_O)	Suggested in experimental studies, not explicitly detected	Yes
Demethylation of MB	Suggested in prior studies, not explicitly studied stepwise	Explicitly modeled

## Data Availability

Data are available from the authors upon reasonable request.

## Abbreviations

The following abbreviations are used in this manuscript:

MB	Methylene blue
ROS	Reactive oxygen species
AOPs	Advanced oxidation processes
BOD	Biological oxygen demand
COD	Chemical oxygen demand
TZVP	Triple-zeta valence polarization
DFT	Density functional theory
PCM	Polarizable Continuum Model
SCF	Self-consistent field
